# Nucleoplasmic Reticulum Formation in Human Endometrial Cells is Steroid Hormone Responsive and Recruits Nascent Components

**DOI:** 10.3390/ijms20235839

**Published:** 2019-11-20

**Authors:** Lior Pytowski, Marek M. Drozdz, Haibo Jiang, Zayra Hernandez, Kurun Kumar, Emily Knott, David J. Vaux

**Affiliations:** 1 Sir William Dunn School of Pathology, University of Oxford, Oxford OX1 3RE, UK; lior.pytowski@path.ox.ac.uk (L.P.); mardrozdz@gmail.com (M.M.D.); zayra.hernandeznunez@path.ox.ac.uk (Z.H.); kurunlkumar@hotmail.co.uk (K.K.); emily.knott@lincoln.ox.ac.uk (E.K.); 2 Weill Cornell Medical College, Cornell University, New York, NY 10065, USA; 3 Centre for Microscopy, Characterisation and Analysis, The University of Western Australia, 35 Stirling Highway, Crawley, WA 6009, Australia; haibo.jiang@uwa.edu.au

**Keywords:** nuclear architecture, nucleoplasmic reticulum, reproductive cycle

## Abstract

The nuclei of cells may exhibit invaginations of the nuclear envelope under a variety of conditions. These invaginations form a branched network termed the nucleoplasmic reticulum (NR), which may be found in cells in pathological and physiological conditions. While an extensive NR is a hallmark of cellular senescence and shows associations with some cancers, very little is known about the formation of NR in physiological conditions, despite the presence of extensive nuclear invaginations in some cell types such as endometrial cells. Here we show that in these cells the NR is formed in response to reproductive hormones. We demonstrate that oestrogen and progesterone are sufficient to induce NR formation and that this process is reversible without cell division upon removal of the hormonal stimulus. Nascent lamins and phospholipids are incorporated into the invaginations suggesting that there is a dedicated machinery for its formation. The induction of NR in endometrial cells offers a new model to study NR formation and function in physiological conditions.

## 1. Introduction

The nuclear envelope (NE) constitutes a boundary between two very different compartments within a cell, the cytoplasm and nucleoplasm. In metazoans, the most prominent structural features of the NE are the outer nuclear membrane (ONM) and the inner nuclear membrane (INM) with a perinuclear space (PNS) between them, which ranges from 30 to 50 nm in thickness [1,2]. Underlying the INM is the nuclear lamina, a proteinaceous meshwork of intermediate filament proteins. The NE is pierced with highly structured nuclear pore complexes (NPC) spanning across the two membranes. These structures are involved in trafficking of molecular cargo in and out of the nucleus [3]. The ONM is connected with the endoplasmic reticulum (ER), thus making the PNS continuous with the ER lumen. Although the INM and ONM meet at the periphery of each NPC, they each retain a fairly distinctive protein composition. The ONM, due to its continuity with the ER is enriched in ER components, while the INM retains its own distinctive array of integral membrane proteins [4]. The NE often shows multiple invaginations of the nuclear membrane into the nucleus, forming an often elaborate network of tubules and INM sheets continuous with the NE. This feature is termed nucleoplasmic reticulum (NR), so named for its structural resemblance to the ER [5,6]. The NR is a widespread feature of many cells and tissues, both under normal cellular conditions [5,6,7,8] and in pathological states [9,10]. These NR structures are classified into two types: type I invaginations involve only the INM with PNS core, and type II involve invagination of both the INM and ONM with cytoplasmic core [11].

The NR appears in many cell types with possibly multiple pathways contributing to its formation. It also occurs as a physiologic cellular response to external stimuli. It has long been recognized that a structurally advanced system of NE invaginations, referred to as the nucleolar channel system (NCS), is a hallmark of the postovulation endometrium [12,13]. Its transient presence manifests in human endometrial cells during a three to four day period during the midluteal, receptive phase of the menstrual cycle [14]. The NCS structure consists of multilamellar cisternae and tubular membrane features within the nucleus that are derived from the INM [12,15]. These cisternae exhibit the presence of NPC proteins and a subset of NE-specific components [14]. The proposed significance of this apparent complex NR is that it is formed in preparation for blastocyst attachment and implantation to the endometrium. This hypothesis is supported by several reports demonstrating the absence or delayed development of NCS in cases of unexplained primary infertility [16,17], further supported by observations that both intrauterine devices and oral contraceptives interfere with NCS formation [18,19]. It has been demonstrated that the formation of NCS can be elicited by the action of oestrogen and progesterone at the time of ovulation [20,21]. Whilst NCS is a unique mix of tubular and cisternal structures, its development from the INM suggests that it may originate as an NR invagination, which, in response to hormones, gains further complexity, possibly representing an advanced NR.

In this work we investigated formation of the NR in an endometrial cell model in response to stimulation with hormones. We used Ishikawa cells, a cell line established from an endometrial adenocarcinoma from a premenopausal patient [22], that express both oestrogen and progesterone receptors. We established a physiological model for studying NR and determined that similar to recently reported NR formation in response to pathological stimulus by prelamin A accumulation [23] the hormonal induction of NR in Ishikawa cells also results in incorporation of newly synthesised membrane phospholipids as well as newly delivered lamina proteins.

## 2. Results and Discussion

### 2.1. Nuclei of Ishikawa Cells Contain NR Structures

**Figure 1:**
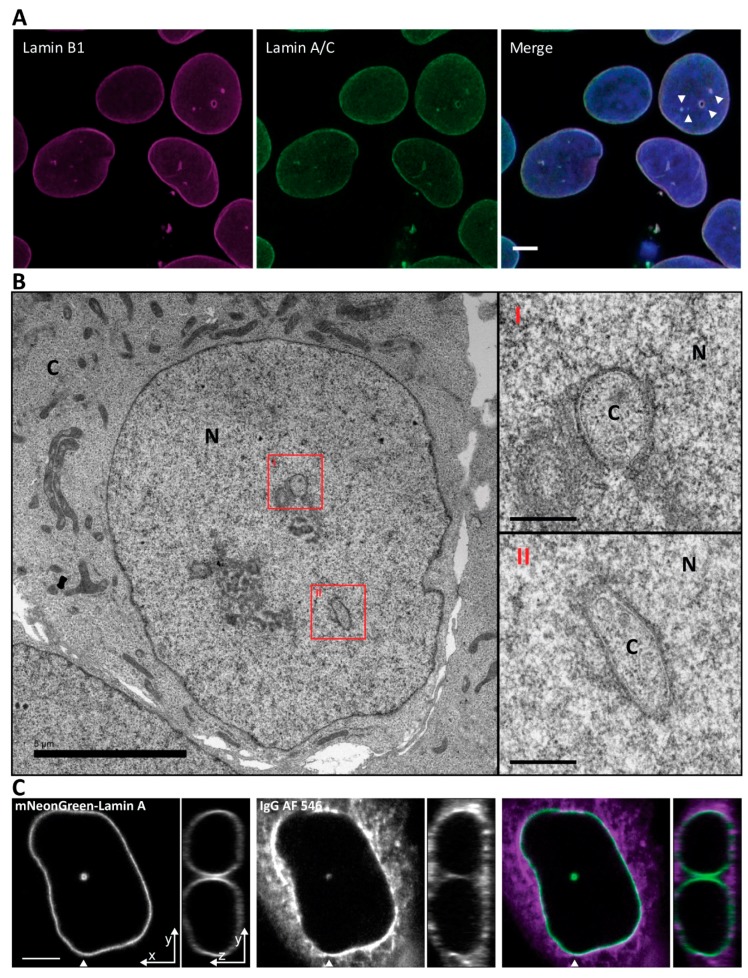
Nuclei of Ishikawa cells contain invaginations of the nuclear envelope forming a nucleoplasmic reticulum. (**A**) Ishikawa cells immunostained with anti-Lamin B1 and anti Lamin A/C. Scale bar 5 µm. (**B**) Electron microscopy micrograph of a high-pressure frozen Ishikawa cell. Scale bar is 5 µm for low magnification nucleus and 0.5 µm for high magnification insets. N, nucleus; C, cytoplasm. (**C**) Cytoplasmic loading by IgG AF 546 to visualize the cytoplasmic core of the invagination. Arrowhead indicates position of orthogonal projection in the panels to the right. Scale bar 5 µm.

Indirect immunofluorescence of fixed Ishikawa cells demonstrated intranuclear foci of both lamin A/C and B marking the NR tubules (Figure 1A). Higher resolution examination by EM microscopy showed that the NR channels present in Ishikawa cells are double membraned and often surrounded by electron dense material including the nuclear lamina and variable amounts of heterochromatin (Figure 1B). Loading of the cytoplasm with a fluorescent 150kDa IgG that is size excluded from the nucleoplasm revealed the cytoplasmic core of the invaginations and is consistent with the presence of INM/ONM double membranes, as is visible in the EM images (Figure 1C).

### 2.2. Formation of NR Tubules in Endometrial Cell Line Is Responsive to Hormones

**Figure 2:**
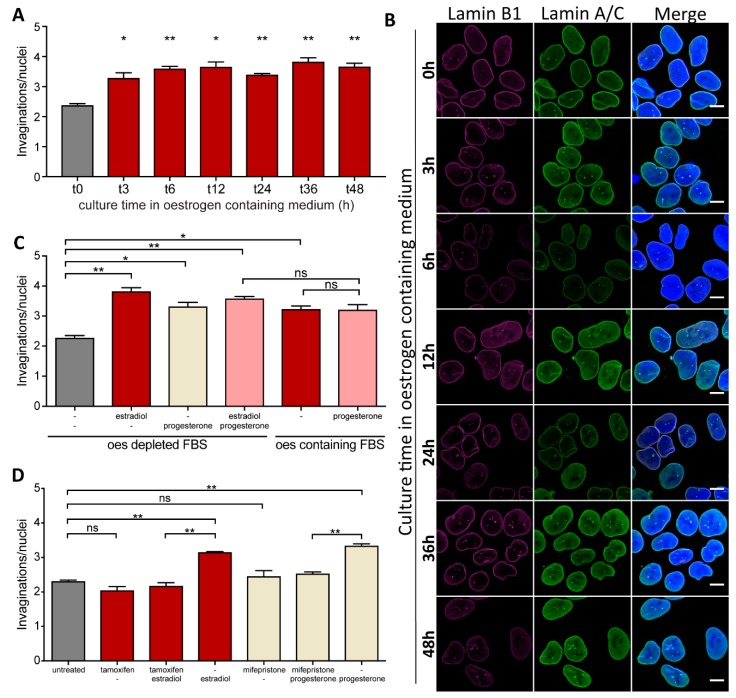
Formation of NR tubules in endometrial cells is hormone responsive. (**A**) NR formation time-course in Ishikawa cells in response to oestrogen. An F-test rejects the null hypothesis (slope = 0) between t0, t3 and t6 (*p* = 0.005) while this null hypothesis cannot be rejected for later time-point (t 12 to t 48) (*p* > 0.5). (**B**) Immunostained Ishikawa cells imaged at different intervals after addition of oestrogen to the culture medium. Scale bar 10 µm. (**C**) NR abundance in Ishikawa cells in response to estradiol and/or progesterone treatment in medium containing either oestrogen-stripped FBS (**oes**trogen-depleted FBS) or regular FBS (**oes**trogen-containing FBS). (**D**) NR abundance in Ishikawa cells treated for 72 h with estradiol or progesterone and their respective antagonists. Data from 2 independent experiments, 100 nuclei each; mean ± SEM; * for *p*-value < 0.05, ** for *p*-value < 0.01, ns for non-significant.

Ishikawa cells cultured in oestrogen-depleted medium show an increase in the mean number of invaginations per nucleus when oestrogen (estradiol) is added to the culture. It is a time-dependent process, and appears to plateau between three and six hours of estradiol treatment (Figure 2A,B). Thus, it suggests that a hormone-sensitive mechanism controlling the complexity of NR exists in these cells and that the effect of oestrogen plateaus by six hours of treatment, with no further NR induction even after prolonged (48 h) hormone exposure. Since the induction of NCS during the menstrual cell cycle requires timely cooperation of both oestrogen and progesterone we tested whether these hormones have added effect in regard to NR formation.

Progesterone added to the cell culture medium, similar to oestrogen, increased the mean number of NR invaginations per nucleus, however there was no synergistic effect of treatment with both hormones (Figure 2C).

These observations remain true for Ishikawa cells cultured in medium containing charcoal-stripped (i.e., hormone-free) FBS to which hormones were then added, and in medium supplemented with regular FBS that normally contains physiologically relevant levels of sex hormones. To further ensure that the increased frequency of NR in Ishikawa cells was due to hormone action on cells, we used tamoxifen and mifepristone that are specific antagonists of oestrogen and progesterone receptors respectively. As expected, co-treatment with either tamoxifen or mifepristone abolished the effect oestrogen or progesterone had on NR abundance in endometrial cells (Figure 2D), suggesting that induction of NR formation was a specific effect of these hormones on the cells.

The data presented here showed that oestrogen or progesterone presence in the culture medium stimulates NR channel formation in Ishikawa cells. It may therefore be speculated that these hormones act on the endometrium in vivo to induce NR proliferation, which plausibly in turn could lead to development of the NCS. This hypothesis could further be supported by the fact that NCS and R-rings (an NCS like structure) originate as type I NR invaginations [15]. Thus, oestrogen and/or progesterone secretion during the menstrual cycle may permit formation of the NCS from NR precursors. We failed, however, to detect any of the more complex structures resembling NCS in our in vitro Ishikawa cell culture despite trying combinations of varying progesterone and oestrogen concentration over 28 days as observed in menstrual cell cycle.

Although the exact mechanism by which oestrogen affects NR proliferation remains speculative, it is possible that the action is mediated through phospholipid synthesis. Oestrogen appears to stimulate the catalytic activity of the rate-limiting enzyme in the Kennedy pathway for production of phosphatidylcholine, CCTα, [24,25] and this enzyme has been demonstrated to play a crucial role in formation of the NR [26,27]. Further work is still needed to fully understand the role of oestrogen in NR formation, including whether or not this is a common response in all steroid-responsive cells.

**Figure 3:**
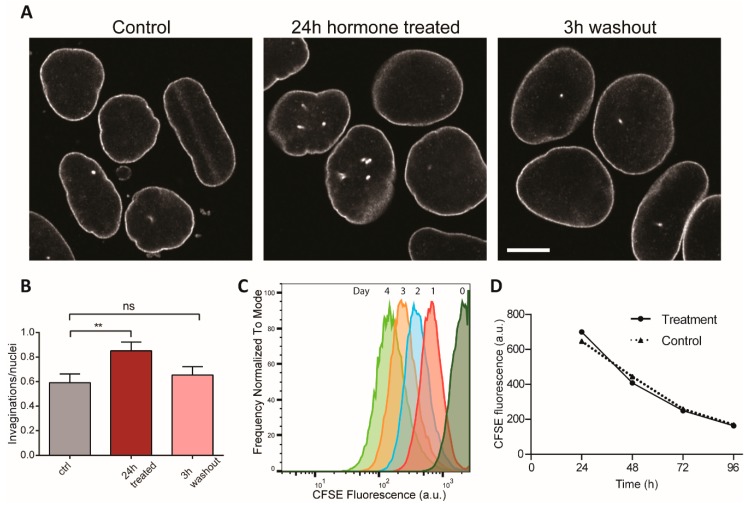
NR formation in endometrial cells is reversible and cell cycle independent. (A) NR induction in Ishikawa cells treated with oestrogen and progesterone for 24h and then subjected to 3 h washout in hormone-free media. Nuclei immunostained with anti-Lamin B1-Cy5. Scale bar 10 µm. (B) NR abundance quantification. Three replicates total, *n* = 242, 262, 203, respectively. ** for *p* < 0.01; ns for non-significant. Error bars represent SEM. (C) Hormone addition or removal does not affect cell division rate. Normalised frequency of CFSE fluorescence of Ishikawa cells measured by flow cytometry. Hormones were added for 48 h then removed for another 48 h. Average cell number per profile = 28,000. Individual flow cytograms in Supplementary Figure S1. (D) Average CFSE loss over time of treated and control samples reveal non-significant difference in cell proliferation rates.

We then addressed whether the invaginations that formed under hormonal induction could be disassembled. We induced NR formation for 24 h with oestrogen and progesterone, then we removed the hormones and counted NR invaginations 3 h later. We found that after hormone removal NR levels rapidly returned to basal level seen prior to induction (Figure 3A,B). To ensure that the loss of NR complexity after hormone removal was not due to cell proliferation, we assessed cell division rates by flow cytometry after 5(6)-carboxyfluorescein N-hydroxysuccinimidyl ester (CFSE) labelling. A CFSE pulse stably labels cells and its fluorescence is halved at every cell division. We found that neither the addition nor the removal of hormones affects the rate of proliferation or the cell cycle stage distribution (Figure 3C and Figure S1). As CFSE fluorescence is halved every 30 h (Figure 3D), the reduction of NR abundance within 3 h of stimulus removal is cell division independent. This indicates that the invaginations of the nuclear envelope are both induced and stabilised during hormone exposure, while hormone removal rapidly reverses these effects and invaginations disappear.

**Figure 4:**
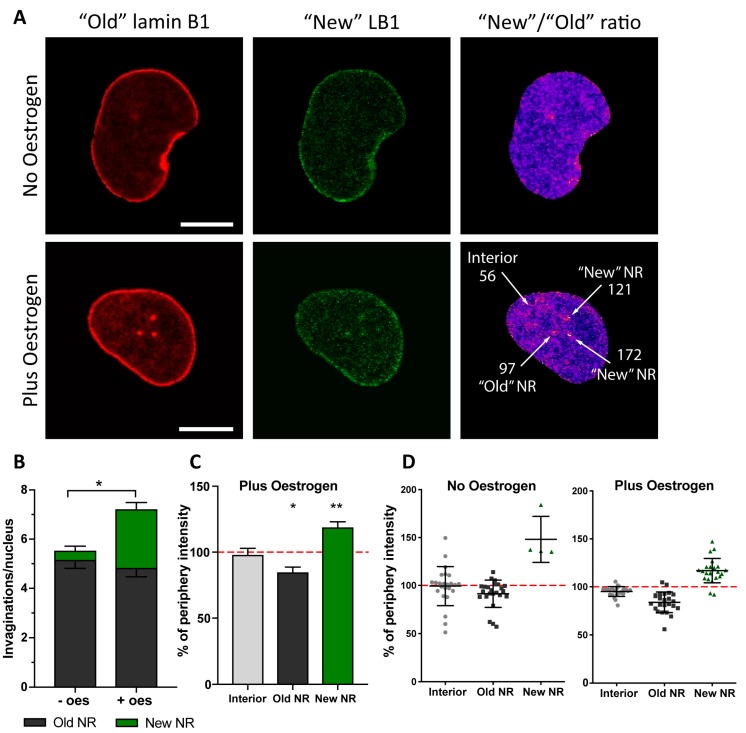
Nascent lamin B1 is incorporated in newly formed invaginations. (**A**) Confocal microscopy of Ishikawa cells expressing lamin B1- Maple3. Indicated are the “old” (red channel) and “new” (green channel) lamin protein pools. Ratiometric image of “New”/”Old” is provided with indication of ratio values for selected ROIs around the features arrowed. (**B**) Evaluation of invagination abundance per nucleus in Ishikawa cells with (+ oes) or without oestrogen (-oes) treatment. (**C**) Pixel intensities of the ROIs defined in based on the ratiometric images and normalised to the signal at the nuclear rim showing increased incorporation of nascent lamin B1 at the newly forming NR channels; results from three independent experiments, 35 cells in total; mean ± SD; ** *p*-value < 0.001; * *p*-value < 0.05. (**D**) An example data plot from a single experiment showing distribution of “New”/”Old” lamin B1 ratio at different nuclear structures and normalised to the nuclear rim ratio with or without oestrogen.

We have recently shown that under pathological conditions, when NR formation is induced by accumulation of abnormally processed lamin A, the newly induced channels and nuclear envelope invaginations require incorporation of nascent lamina proteins as well as newly synthesised phospholipids [23]. We decided to test whether NR formed in Ishikawa cells in response to a physiological stimulus exhibited the same property. To monitor incorporation of newly synthesised lamins to the nuclear envelope during NR induction with oestrogen, similar to previous work, we expressed lamin B1 tagged with photoconvertible fluorescent protein Maple3 in Ishikawa cells. The lamin B1 Maple3 tag was fully photoconverted from a green into a red fluorescent protein by exposure to 405 nm monochromatic light and thus marked the pool of “old” lamin B1, pre-existing in a cell prior to photoconversion (Figure 4A). After a recovery period of 18 h, cell culture medium was supplemented with oestrogen and induction of NR followed for 7–9 h. Then the pool of lamin B1 synthesised post-photoconversion was imaged in green channel (“new” lamin B1), while previously photoconverted protein (“old” lamin B1) was simultaneously recorded in red channel, which allowed for measuring the ratio of nascent lamin B1 (expressed within past 25–27 h) relative to lamin B1 present in a cell prior to photoconversion. ROIs were applied to ratiometric images for analysis of pixel intensities that were further normalised to the nuclear rim intensities in that cell.

As observed earlier, oestrogen treatment for 7–9 h increased number of detected NR channels. More importantly though, and similarly to a pathological model we reported earlier, newly formed NR in the endometrial cell model showed significant enrichment in nascent lamin B1 (Figure 4B), and incorporated newly synthesised protein at much higher rate than the bulk nuclear envelope or pre-existing NR (Figure 4C,D). Interestingly, a few cells in the control group without hormone stimulation also formed new NR tubules enriched in nascent lamin B1 during the experiment (Figure 4D). Although the majority did not, this is an observation similar to that which we observed in control samples in the pathological model of NR induction by prelamin A accumulation [23].

**Figure 5:**
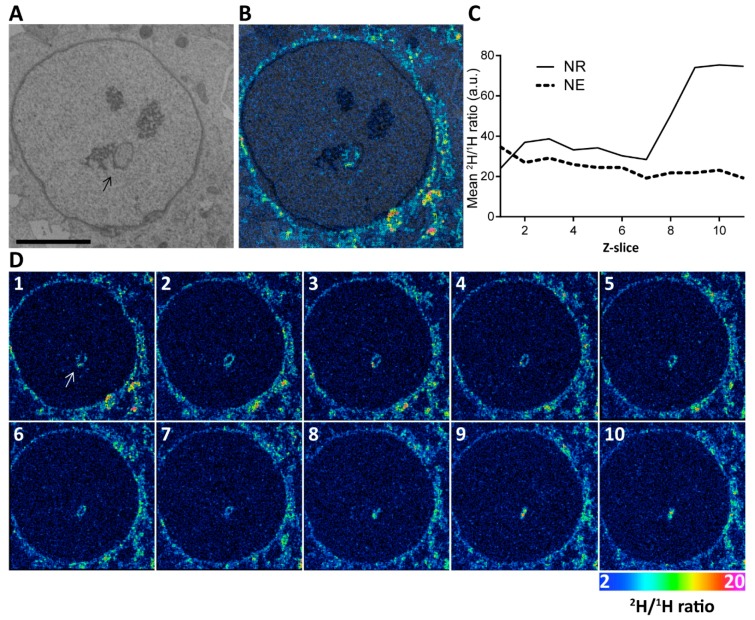
Nascent phospholipids are incorporated in the forming NR during estradiol stimulation. (**A**) Representative backscattered electron micrograph of an Ishikawa nucleus pulse labelled with deuterated choline. Arrow points to an NR channel. Scale bar 5 µm. (**B**) Overlay of the electron micrograph and NanoSIMS image showing deuterium enrichment at the invagination. (**C**) Quantification of the mean ^2^H/^1^H ratio in the NR versus the NE rim for the ion-beam eroded z-series through this cell. The average ^2^H/^1^H ratio for two regions of interest (total NE and NR) are shown for each z plane. This shows increasing nascent deuterated choline signal in the tip of the NR invagination where no lumen is visible. See also Supplementary Movie S1. (**D**) Panel of individual sections of 3D reconstruction. Color scale of NanoSIMS images 2–20 equals 0.02–0.2% of ^2^H/^1^H ratio.

Since CCTα, the rate limiting enzyme of phosphatidylcholine production, is oestrogen responsive and largely if not exclusively confined to the nucleus [28], we next looked into the distribution of nascent phospholipids during NR induction in our physiological model. Here cells were pulsed with deuterated choline for 12 h in the presence of estradiol, before being fixed and prepared for NanoSIMS analysis. Cells were imaged by backscattered electron microscopy and correlated to the NanoSIMS analysis (Figure 5A,B). While foci of high deuterium incorporation were observed along the walls of NR channel as seen in the images of individual planes, the highest level of deuterium enrichment was detected at the blind-ended NR tip where the channel no longer has a lumen. This distribution was confirmed by measuring the average intensity of deuterium signal at the NE or NR in the depth profile (Figure 5C,D; Supplementary Movie S1). Similar enrichment of nascent phospholipids at forming NR was also observed previously in a pathological model of NR induction [23].

Taken together, the results presented here establish a physiologically relevant model for studying NR induction and resolution. Moreover, the involvement of nascent protein and lipid in the formation of hormone-induced NR structures is consistent with a model of NR biogenesis in which the structure develops de novo rather than resulting from deformation of a pre-existing NE by external forces, whether from within or without. This is similar to recent observations on pharmacological induction of a pathological NR by accumulation of prelamin A [23].

The observation of rapid loss of induced NR after washout implies that a tonic signal from hormones maintains NR structures, and opens new avenues for analysis of NR regulation. In particular, these data already confirm that the involution of NR structures may occur physiologically without the intervention of mitosis, as had already been demonstrated for NR induction [26]. Future experiments correlating the timing of gene expression, protein delivery and post-translational modification with the involution of NR structures upon hormone withdrawal offers an avenue further to identify signals that regulate abundance of this nuclear structure. Furthermore, it is possible that the simple model described here may recapitulate important features of the hormone-dependent changes that prepare the endometrium for implantation. If this is the case, NR formation in cultured endometrial cells may offer a useful surrogate measure in studies of clinical and pharmacological interventions to enhance or prevent fertility.

## 3. Materials and Methods

### 3.1. Mammalian Cell Culture, Transfection and Treatments

Ishikawa cells (ECACC 99040201) were cultured in Dulbecco’s Modified Eagle Medium (DMEM) supplemented with 10% charcoal stripped foetal calf serum (FCS, F6765, Sigma, Saint Louis, MO, USA), 1% non-essential amino acids, and penicillin and streptomycin antibiotics (100 units/mL). Cells were transfected with Lipofectamine 2000 (Invitrogen, Carlsbad, CA, USA) following the manufacturer’s instructions. Hormones were added to the media for the time indicated. Estradiol (Sigma) was used at 150 pg/mL and progesterone (Sigma) at 10 ng/mL. Mifepristone (Sigma) and tamoxifen (Sigma) were used at 1 µM concentration.

### 3.2. Generation of mNeonGreen-LmnA Cell Line by CRISPR-Cas9

mNeonGreen was inserted after the start codon of LmnA using a double-nicking strategy with the Cas9 D10A nickase mutant. The donor plasmid for LmnA consisted of the insert enclosed in a 475 bp C term homology arm and a 483 bp N term homology arm between positions 120 and 121 of the lacZa gene of pBluescript. The donor plasmids were assembled by isothermal assembly (NEBuilder^®^ HiFi DNA Assembly, New England BioLabs, Ipswich, MA, USA). *gRNA plasmid generation***:** gRNA and plasmid design were done following the recommendations of Ran et al. (2013) [29]. Guides were designed with the MIT design tool (Zhang Lab, MIT, MA, USA): http://crispr.mit.edu/ and inserted into Addgene Plasmid #48141 pSpCas9n(BB)-2A-Puro (PX462, a gift from Feng Zhang, MIT, MA, USA). Guide RNAs were 5’-ACGGGGTCTCCATGGCCGGCAGG-3’ and 5’-AGCGGCGCGCCACCCGCAGCGGG-3’.

### 3.3. Transfection and Cell Sorting

Plasmids containing the guides and donor templates were transfected in HeLa cells using Lipofectamine 2000 following the manufacturer’s recommendations. Cells were then left to recover and to express the fusion protein for a week before proceeding with cell sorting. Cell sorting was done on a BD FACS Aria III (BD Biosciences, San Jose, CA, USA) run with the BD FACSDiva software version 8.0.2 (BD Biosciences); positive cells were sorted if green or red fluorescence was 10^3^ times brighter that control cells. Positive cells were single cell sorted in an optical 96 well plate containing preconditioned media and adequate localisation of the fusion protein was confirmed by confocal microscopy and later by Sanger sequencing to confirm correct insertion of the mNeonGreen cassette (Allele Biotechnology, San Diego, CA, USA).

### 3.4. Immunofluorescence and Confocal Microscopy

Cells were seeded on glass coverslips in 24 well plates. Cells were fixed in 4% PFA for 10 min and subsequently permeabilized for 5 min with 0.5% X-100 Triton. Cells were blocked in 0.5% skin fish gelatine for 1 h. Antibodies were diluted in the blocking solution. Primary antibodies were goat anti-lamin B1 (C-20, Santa Cruz Biotechnology, Santa Cruz, CA, USA), and mouse anti-lamin A/C (4C11) Active Motif (Carlsbad, CA, USA). Secondary antibodies used were donkey anti-mouse and donkey anti-goat conjugated to Alexa Fluor 488 and Alexa Fluor 647 (Invitrogen, Waltham, MA, USA). Cytoplasmic loading was conducted by permeabilising live cells with a 750 μg/mL solution of saponin containing a secondary goat anti rabbit IgG Alexa Fluor 546 (80 μL stock solution per 1mL of detergent solution) at 4 °C for 5 min then fixed immediately with cold 4% PFA. Samples were imaged immediately. Fixed cells were imaged on the LSM5 Zeiss Inverted 510 META laser scanning microscope (Zeiss, Oberkochen, Germany) using a Plan Apo 63 × 1.4 NA oil immersion lens. Images were acquired using Zen2009 operating software (Zeiss). Samples of cytoplasmic loading and the hormone removal experiment were imaged on a Zeiss LSM880 Airyscan using a Plan Apo 40 × 1.3 NA oil immersion lens. Images were acquired and pre-processed using ZenBlack (Zeiss). Collected images were analysed in FIJI [30].

### 3.5. Live Cell Microscopy of Photoconvertible Maple3-Lamin B1

This experiment was conducted as described previously [23]. Briefly, cells were seeded in optical 24 well plates 24 h before transfection. Cells were left for another 24 h to express the fusion protein and were then imaged on Olympus FV1200 laser scanning microscope (Olympus, Tokyo, Japan) equipped with temperature and CO_2_ chamber for live cell work. 60 × 1.4 NA oil objective was used and images were acquired with Fluoview software (Olympus, Tokyo, Japan). Maple3 photoconversion was done by exposing cells to UV light for 60 sec at 12% lamp power (U-HGLGPS 100 W mercury lamp). After a recovery period of 18 h, cell culture medium was supplemented with oestrogen for 7–9 h and cells were then imaged. Ratiometric images were generated by dividing the green channel intensity (“new lamin”) by the red channel intensity (“old lamin”).

### 3.6. Flow Cytometry

Adherent cells were stained with 5 µM CFSE (BioLegend, San Diego, CA, USA) for 15 min. Excess CFSE was removed by a 2 min wash in PBS. Cells were then trypsinised and reseeded in 10 flasks–one for each time point plus controls. At the appropriate time, cells were harvested by trypsinisation and fixed in 4% PFA. CFSE fluorescence was measured in a BD Fortessa X20 flow cytometer (BD Biosciences). Results were analysed with FlowJo software (FlowJo, Ashland, OR, USA).

### 3.7. Correlative Backscattered Electron Microscopy and NanoSIMS Imaging

Deuterium labelling of phospholipids and sample processing was performed as described elsewhere [23]. Briefly, prior to labelling Ishikawa cells were cultured in low serum (1%) media for 24 h. Then deuterated choline chloride d_9_ (CDN Isotopes, Pointe-Claire, QC, Canada) was added at 80 mM, together with 150 pg/mL estradiol for 12 h. Upon treatment completion, cells on coverslips were washed with PBS and fixed in 4% paraformaldehyde and 1% glutaraldehyde in 100 mM PIPES pH 7.4, followed by secondary fixation in 2.5% glutaraldehyde in 100 mM PIPES pH 7.4. Osmication, dehydration and gradual infiltration with Agar 100 epoxy resin (Agar Scientific, Stansted, UK) followed. The cells were embedded as a monolayer in the resin and semi-thin sections were cut for further analysis. Backscattered electron (BSE) imaging was performed using the NVision FIB scanning electron microscope and BSE images were acquired with a 2 kV incident beam with a standard aperture (30 µm) and 5-mm working distance.

Upon the completion of BSE imaging, the sections were coated with 5 nm of platinum in a Cressington 208HR high-resolution sputter coater (Cressington Scientific Instruments Ltd., Watford, UK) to provide the surface conductive for Nanoscale Secondary Ion Mass Spectrometry (NanoSIMS) imaging on Cameca NanoSIMS50 (Cameca, Gennevilliers, France).

First, the Cs^+^ primary beam was used to remove the platinum on the surface at selected locations, simultaneously implementing a Cs^+^ dose of 1.0 × 10^17^ ions/cm^2^. Small apertures (D1 = 3 or D1 = 4) were used for imaging a single cell in order to match the size of primary beam to the pixel size. The instrument was tuned for ^2^H^–^ and ^1^H^–^ to allow calculation of the ^2^H/^1^H ratio. The NanoSIMS images were acquired with a dwell time of 30,000 µs per pixel for 256 × 256 pixel images. A median filter with radius of 3 pixels was applied to the Hue Saturation Intensity (HSI) image. A single NanoSIMS image corresponds to ~10 nm specimen thickness. The BSE and NanoSIMS images were aligned, and the local ^2^H/^1^H ratio quantified in FIJI software. Data from ImageJ was then imported to Excel and GraphPad Prism for further analysis

### 3.8. Statistics

Unless indicated otherwise, results represent at least three independent biological replicates. Scatter plots show pooled data, while bar graphs represent the means with standard deviation of the results from independent repeats. Exact number of repeats and cells in a cohort is indicated in figure legends. A two-tailed, unpaired Student’s t-test was employed to determine statistical significance of the results.

## Supplementary Materials

Supplementary materials can be found at https://www.mdpi.com/1422-0067/20/23/5839/s1.
