# Supplementary material for: Nucleoplasmic Reticulum Formation in Human Endometrial Cells is Steroid Hormone Responsive and Recruits Nascent Components
Source: Int J Mol Sci. 2019 Nov 20;20(23):5839. doi: 10.3390/ijms20235839 (PMC6929123; doi:10.3390/ijms20235839)
Supplement: Supplementary file 1 [file ijms-20-05839-s001.pdf]

Supplementary materials:

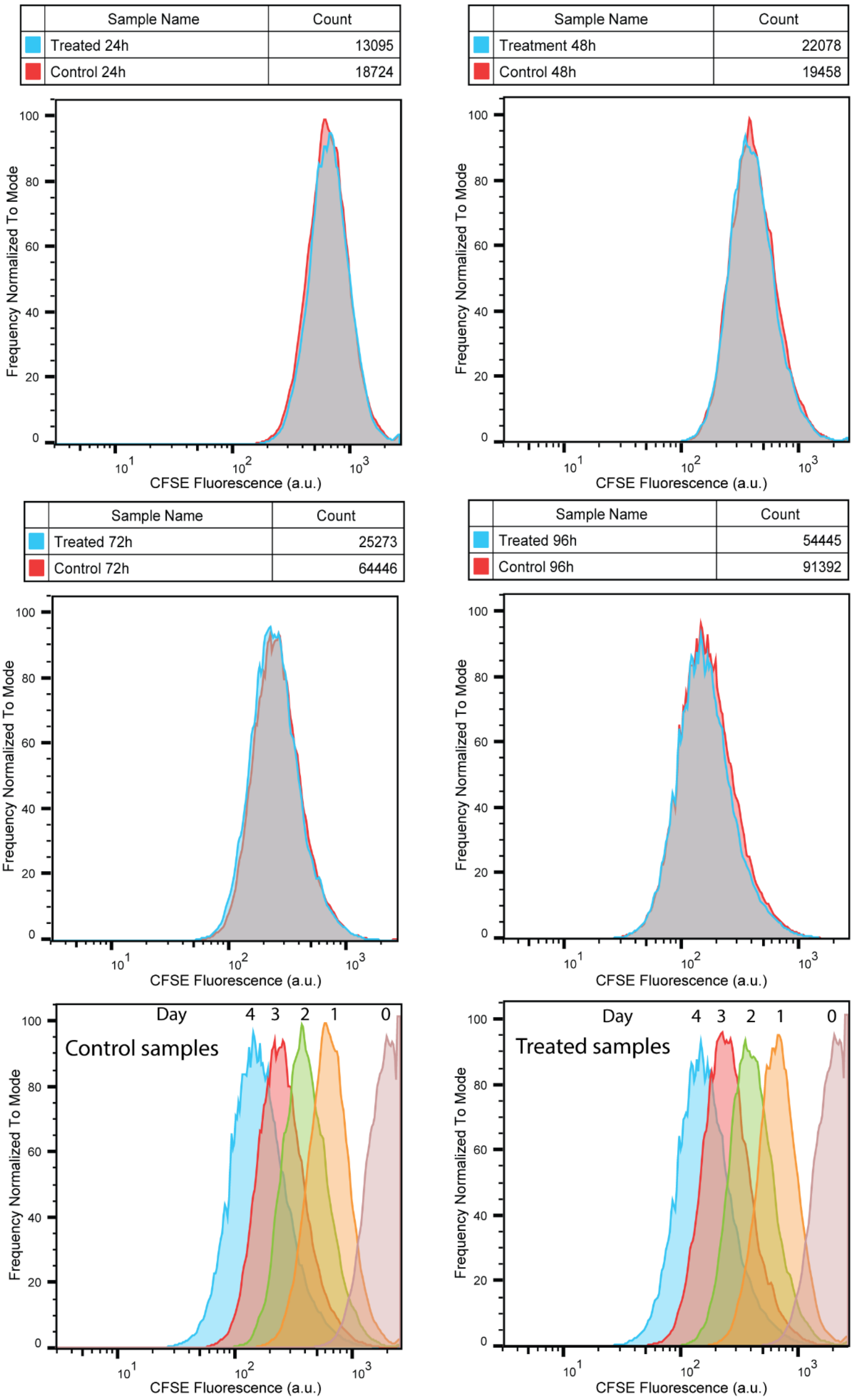

Figure S1. Hormonal exposure and removal during Ishikawa cell culture does not affect cell division rate. Flow cytograms of cells loaded with CFSE at t=0 and exposed to oestrogen and progesterone for 48 hours. After the initial 48, hormones were removed for another 48 hours. CFSE intensity per cell was measured by flow cytometry at 24 hour intervals during this experiment.

Supplementary Movie 1: 3D NanoSIMS analysis of nascent phospholipid distribution in Ishikawa cell after pulse-chase labelling with deuterated choline. Movie corresponds to Figure 5
